# Excitability of jcBNST Neurons Is Reduced in Alcohol-Dependent Animals during Protracted Alcohol Withdrawal

**DOI:** 10.1371/journal.pone.0042313

**Published:** 2012-08-21

**Authors:** Attila Szücs, Fulvia Berton, Pietro Paolo Sanna, Walter Francesconi

**Affiliations:** 1 BioCircuits Institute, University of California San Diego, La Jolla, California, United States of America; 2 Balaton Limnological Research Institute of the Hungarian Academy of Sciences, Tihany, Hungary; 3 Molecular and Integrative Neurosciences Department, The Scripps Research Institute, La Jolla, California, United States of America; 4 Department of Biology, University of Pisa, Pisa, Italy; Centre for Addiction and Mental Health, Canada

## Abstract

Alcohol dependence and withdrawal has been shown to cause neuroadaptive changes at multiple levels of the nervous system. At the neuron level, adaptations of synaptic connections have been extensively studied in a number of brain areas and accumulating evidence also shows the importance of alcohol dependence-related changes in the intrinsic cellular properties of neurons. At the same time, it is still largely unknown how such neural adaptations impact the firing and integrative properties of neurons. To address these problems, here, we analyze physiological properties of neurons in the bed nucleus of stria terminalis (jcBNST) in animals with a history of alcohol dependence. As a comprehensive approach, first we measure passive and active membrane properties of neurons using conventional current clamp protocols and then analyze their firing responses under the action of simulated synaptic bombardment via dynamic clamp. We find that most physiological properties as measured by DC current injection are barely affected during protracted withdrawal. However, neuronal excitability as measured from firing responses under simulated synaptic inputs with the dynamic clamp is markedly reduced in all 3 types of jcBNST neurons. These results support the importance of studying the effects of alcohol and drugs of abuse on the firing properties of neurons with dynamic clamp protocols designed to bring the neurons into a high conductance state. Since the jcBNST integrates excitatory inputs from the basolateral amygdala (BLA) and cortical inputs from the infralimbic and the insular cortices and in turn is believed to contribute to the inhibitory input to the central nucleus of the amygdala (CeA) the reduced excitability of the jcBNST during protracted withdrawal in alcohol-dependent animals will likely affect ability of the jcBNST to shape the activity and output of the CeA.

## Introduction

The neuroadaptive changes associated with alcohol and drug dependence and withdrawal have been a major focus of neuroscience research. Accumulating evidence suggests that the functional, systemic level effects of alcohol dependence arise from complex interactions and adaptations at multiple levels in the nervous system. While neuroadaptive changes of synaptic properties induced by alcohol and drugs of abuse have been extensively studied in the past years [1], [2], [3], [4], [5], [6], alterations of voltage-dependent currents have been also shown to play potentially important roles in the functional effects associated with alcohol and drug dependence [7], [8], [9], [10], [11]. However, a better understanding of how such cellular and subcellular level adaptations translate to the firing of neurons and integration of synaptic inputs remains largely unknown. In the present study we perform an analysis of passive membrane properties, intrinsic excitability and integrative properties of neurons of the juxtacapsular nucleus of the bed nucleus of stria terminalis (jcBNST) in animals with a history of alcohol dependence.

The lateral BNST is among the brain regions that compose the extended amygdala together with the central (CeA) and medial (MeA) nuclei of the amygdala, among others [12], [13]. The jcBNST is located in the anterolateral BNST that also includes the rhomboid and oval nuclei. The anterolateral BNST has been implicated in modulating autonomic responses [14] and in the adaptations induced by alcohol and drugs of abuse [15], [16], [17]. However, the connection pattern of the jcBNST sets it aside from the other anterolateral nuclei of the BNST. In fact, it is the only nucleus in the anterolateral BNST that does not receive inputs from the CeA [18], [19]. The jcBNST receives robust excitatory efference from the BLA and the amygdalopiriform transition area through the stria terminalis and ansa peduncularis [20], [21], [22] as well as projections form the dysgranular insular cortex, and the infralimbic cortex [20], [22], [23], [24] and, in turn, sends a major GABAergic projection to the medial part of the central nucleus of the amygdala (CeAm) [23], [25]. Since the jcBNST lacks cells expressing glutamatergic markers [26] and has abundant GABAergic cell bodies [27], [28] its projections are likely inhibitory. Therefore the jcBNST is well positioned to integrate excitatory inputs form the BLA and dysgranular insular and infralimbic cortical regions and to contribute inhibitory control over the CeA. Additionally, the jcBNST can also indirectly influence the CeA through its projections to the basolateral amygdala (BLA) and other cell groups that in turn send projections to the CeA [21], [23], [25]. Thus changes in the integration properties of jcBNST neurons may contribute to the overall amygdala output and to the persistent emotional dysregulation associated with protracted withdrawal.

We have previously shown that protracted withdrawal from alcohol, cocaine, and heroin as well as chronic intracerebroventricular treatment with corticotropin-releasing factor (CRF) induce common functional adaptations in the jcBNST characterized by the impairment of a form of plasticity of intrinsic excitability [29]. Here we study excitability and other physiological properties of jcBNST neurons in animals with history of alcohol dependence during protracted withdrawal. In particular we investigate neuronal excitability using either standard intracellular injection of direct current (DC) or with the injection of artificial synaptic conductances with the dynamic clamp method [30], [31]. DC current injection is a method traditionally used to reveal factors that regulate neural firing behavior. However, the simple current step stimulus does not resemble the complex synaptic current inputs neurons experience in their natural synaptic environment [32]. Neural excitability *in vivo* is determined by interactions between intrinsic membrane properties and synaptic inputs. Hence, the injection of artificial synaptic conductances offers a valuable alternative to study neuronal firing and excitability under temporally complex synaptic bombardment like neurons experience *in vivo* [33].

Interestingly, using synaptic stimulation via dynamic clamp, we observe a significant reduction of intrinsic excitability of jcBNST neurons from alcohol-dependent rats that is not evident by using DC current injection. This observation suggests that adaptations induced by alcohol and drug abuse may involve neural mechanisms that are sensitive to the dynamics of the input. The reduced excitability of jcBNST neurons in animals with a history of alcohol dependence is likely to result in reduced inhibition of the CeAm, the main output nucleus of the amygdala [34]. Since the CeAm is believed to be an important output nucleus of the amygdala for the expression of emotional responses [35], [36], the reduced inhibitory input from the jcBNST may contribute to the negative affective state that characterizes protracted abstinence in post-dependent individuals.

## Results

As a general strategy, we performed experiments on 3 types of jcBNST neurons from control and alcohol-dependent animals using either conventional current step (DC) stimulation or simulated synaptic inputs via dynamic clamp. Injection of constant current steps of variable amplitude departing from I = 0 allowed us to assess the neurons overall physiological properties. On the other hand, the dynamic clamp experiments allowed us to investigate how the temporal structure of spike responses of jcBNST neurons depended on their intrinsic cellular properties as well as the strength of the simulated synaptic inputs.

### Physiological properties of three types of jcBNST neurons

**Figure 1:**
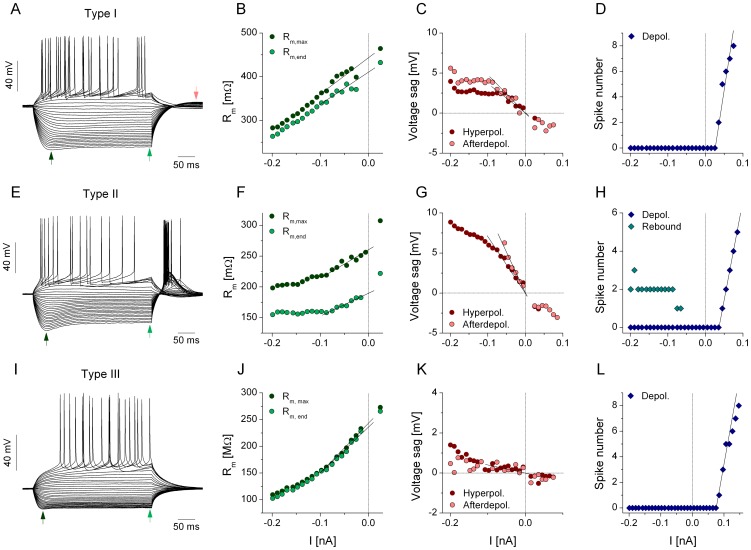
Analysis of physiological properties of 3 types of jcBNST neurons. Current steps of 350 ms duration start at −200 pA and the DC level is increased by 10 pA in the successive pulses. The voltage responses of three types of jcBNST neurons are shown in the left column. The type I neuron in A displays moderate voltage sag during hyperpolarization. The maximal voltage deflection (V_max_, left arrow) and the voltage at the end of the pulse (V_end_, right arrow) are used to build the input resistance curves on B. Panel C shows the voltage sag that is observed during the hyperpolarizing step and during the afterdepolarization (see red arrow in A). The spike number as a function of the input current is shown in D. Here the rheobase and the initial slope of the spike number curve are determined. The type II neuron shown in E exhibits a strong voltage sag and post-inhibitory rebound firing. For this type of neuron the two resistance curves strongly deviate (F). The number of spikes under the depolarization and under the PIR response is shown in H. The type III neuron demonstrated in I exhibits a strong inward rectification but no voltage sag is observed. The input resistance at −0.2 nA is less than half of that measured at resting membrane potential (extrapolated using linear regression, J). The voltage sag is nearly zero in the range of applied current steps (K). The rheobase of this neuron is significantly higher than that of the previous two types (L).

Using voltage responses of jcBNST neurons from DC step experiments we extracted several physiological parameters that were useful both for classification [37], [38], [39] of the neurons but also to reveal potential alcohol withdrawal-induced changes in their biophysical properties. There are three main types of jcBNST neurons that can be identified in DC current step experiments. In particular, type I neurons display moderate voltage sag during hyperpolarization suggestive of the expression of I_h_ (Fig. 1A, B, C, D). Type II neurons (Fig. 1E, F, G, H), as a distinctive feature, produce post-inhibitory spikes in response to preceding negative current steps. Another characteristic of such neurons is the stronger voltage sag than in type I neurons as shown in Fig. 1G. Finally, perhaps the most readily identifiable cell type is the type III neuron that has a high rheobase, and prominent inward rectification suggestive of the expression of the inwardly rectifying K-current I_KIR_ (Fig. 1I, K, L). These neurons typically start firing in response to stronger depolarizing currents (0.1 nA and above) and their first spike appear after a characteristic slow voltage ramp along the stimulus step (Fig. 1I). Figure 1 demonstrates the various steps of calculations to obtain the physiological parameters we analyzed in our study. As a common feature of all jcBNST neurons, their input resistance depends on the applied current due to the effect of inward rectification (type III, Fig. 1J) or voltage sag (type I and II, Fig. 1B, F). Here we use linear fit to extrapolate the input resistance at rest (I = 0). Next, we plot the voltage sag as a function of the input current (Fig. 1C, G, K). In type I and II neurons the voltage sag grows linearly as the hyperpolarizing current increases (from 0 to −70 pA) and then the sag curve flattens (see Fig. 1C, G). We use the initial slope of the voltage sag curve as a parameter characterizing the degree of sag depolarization. In addition to the features obtained from the subthreshold voltage traces, we analyze the suprathreshold behavior of the neurons. We count spikes separately during the depolarizing current steps and after the hyperpolarizing current steps when post-inhibitory firing occurs (Fig. 1D, H, L). Spike number of firing responses under depolarization is nearly a linear function of the input current when using moderate current levels. We use the slope of the fitted linear function (gain, I–N slope) as a quantitative measure of the excitability of the neuron under DC step stimulation. Another parameter that characterizes the excitability is the rheobase. This one is obtained by reading the current level where the fitted line departs from zero.

A summary of 6 physiological parameters of the 3 types of jcBNST neurons is shown in Table 1. Comparing their resting membrane potential we find that cell type II neurons tend to be more depolarized than the others while type III neurons are the most hyperpolarized ones. Furthermore, this latter cell type has the lowest input resistance (R_max_ at I = 0) and the highest rheobase. Also, cell type specific differences are found in the amount of voltage sag, slope of the input resistance curves, spike threshold and other parameters.

**Table 1:** Comparison of physiological properties of jcBNST neurons across cell types and treatment groups.

Cell type	N	Resting V_m_ mV	Resistance MΩ	Sag slope mV/nA	Rheobase pA	I–N slope pA^−1^	Spike thresh. mV
I _Cont_	20	−61.4±0.7 [−65.9 : −51.1] II, III	337±31 [147 : 566] III	−38.7±3.9 [−80.2 : −17.5] II	56.7±7.1 [15 : 117] II, III	106±13 [35 : 226] III	−35.0±0.8 [−41.3 : −27.8] II
I _EtOH_	12	−61.7±1.4 [−73.2 : −54.8] II, III	353±28 [204 : 563] III	−41.5±5.2 [−89.4 : −24.9] II	50.9±7.7 [30 : 127] II, III	95±13 [16 : 171]	−37.0±0.9 [−44.4 : −34.1]
II _Cont_	18	−56.8±0.7 [−61.3 : −50.6] I, III	399±33 [192 : 664] III	***−95.3±7.6*** [−154 : −52.4] I	30.9±4.1 [2 : 65] I, III	126±26 [54 : 514]	−38.2±0.6 [−44.0 : −33.6] I, III
II _EtOH_	10	−55.3±1.2 [−60.5 : −48.9] I, III	427±36 [258 : 608] III	***−66.9±9.5*** [−109 : −8.3] I	27.8±3.8 [10 : 45] I, III	167±29 [42 : 305]	−36.2±0.9 [−39.9 : −30.7]
III _Cont_	31	−72.0±0.9 [−81.6 : −57.2] I,II	218±20 [72 : 536] I, II	N/A	119.8±9.0 [29 : 219] I, II	164±12 [71 : 312] I	−35.0±0.7 [−42.1 : −27.4] II
III _EtOH_	16	−74.2±0.8 [−80.6 : −70.2] I, II	223±23 [59 : 389] I, II	N/A	115.8±13.8 [58 : 248] I, II	183±31 [63 : 444]	−35.1±0.7 [−40.3 : −31.3]

Values are expressed as means ± SEM. The sample minimum and maximum are shown in brackets. Cell type labels under the values represent statistically significant differences from the corresponding types either in control or EtOH groups. Values marked in bold-italic represent significant differences between parameters of control and EtOH cells belonging to the same type. One-way ANOVA with Bonferroni test for means comparison was used for the parameters of the three types of cells from either the control or EtOH groups. Two-sample t-tests were performed for control-EtOH comparison of identical types of neurons (p = 0.05, numbers of cells are shown under N).

### Effects of alcohol withdrawal on physiological properties of jcBNST neurons

The physiological parameters we extracted from the DC step experiments were indicators or correlates of various intrinsic properties such as cell type specific expression of voltage-gated membrane conductances (e.g. I_h_, I_KIR_). As a first observation from the comparative analysis, we found that protracted withdrawal did not induce significant alterations in most physiological parameters of the jcBNST neurons. In particular, the resting membrane potential, input resistance, rheobase, spike threshold and other features of the neurons were affected below the level of statistical significance in all three types of neurons (Table 1). Conversely, the mean slope of the voltage sag curve (as measured under hyperpolarizing current steps) in type II neurons was significantly reduced by 30% when comparing normal and neurons from alcohol dependent rats (Table 1). This finding suggests that protracted withdrawal might alter the operation of hyperpolarization-activated nonspecific cation conductances (I_h_) in type II neurons. As our data from control neurons show, the slope of voltage sag is the highest in type II neurons (Table 1), suggesting the abundance of I_h_ in these neurons. The voltage sag of type I neurons - that is about half the size of that in type II neurons - was unaffected by alcohol dependence during protracted withdrawal (Table 1). While I_h_ was not directly measured in our experiments, we found that application of a specific blocker of I_h_ ZD7288 reliably eliminated the voltage sag in type II as well as in type I neurons (n = 5), confirming that the voltage sag and the amount of I_h_ in the jcBNST neurons are strongly correlated.

Type II neurons, and to a lesser extent type I neurons, display a characteristic afterdepolarization at the termination of hyperpolarizing current steps. Here, the membrane potential temporally repolarizes to a level that is more positive than the resting membrane potential. Hence, one can compare the amplitude of afterdepolarization across neurons and treatment groups. While the sag under hyperpolarization in type II neurons was significantly decreased by alcohol withdrawal, the slope of afterdepolarization curve was moderately increased from −96.1±9.5 mV/nA (n = 11) to −131.1±18.2 mV/nA (n = 10) mV/nA, expressed as mean ± S.E. However, this effect was not significant (p<0.11).

### Firing dynamics of jcBNST neurons under the action of simulated synaptic inputs in control and alcohol-dependent rats during protracted withdrawal

**Figure 2:**
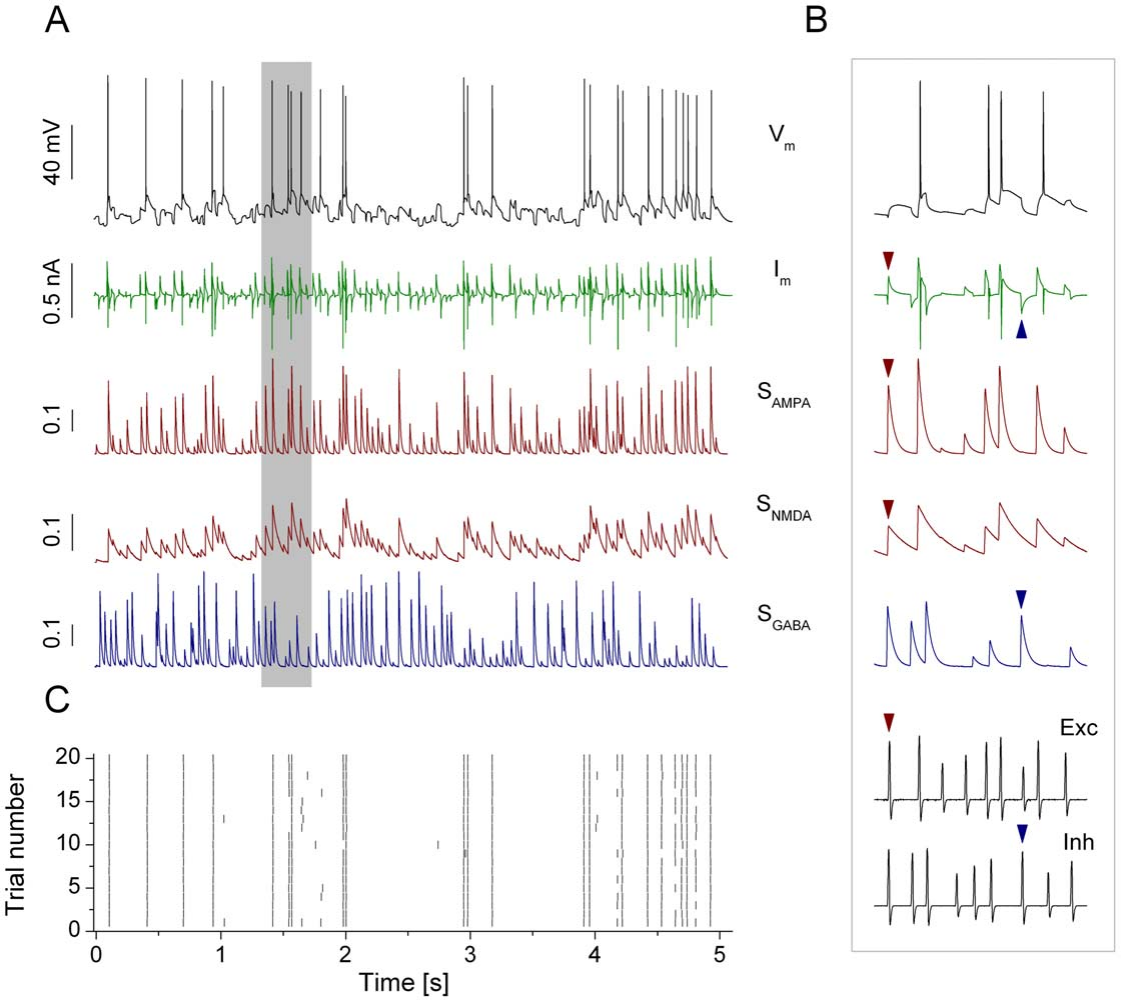
jcBNST neurons fire reliably and with high precision when receiving conductance inputs via dynamic clamp. The membrane potential trace of a type II neuron during one presentation of the 5 s stimulus and the input waveforms are shown in A. The green trace under the voltage response is the current (I_m_) that is generated by the dynamic clamp system. The synaptic activation functions for the AMPA-, NMDA- and GABA-type inputs are displayed below (S_AMPA_, S_NMDA_ and S_GABA_, all unitless with 0 as baseline). A brief section (gray bar) of the voltage response and the input waveforms is zoomed and shown in the right (B). Separate excitatory and inhibitory (Exc and Inh, bottom traces) voltage waveforms are used to elicit barrages of EPSCs and IPSCs in the neuron. A selected ‘spike’ in the excitatory waveform, the corresponding AMPA- and NMDA-type conductance transients and the injected EPSC are indicated by red triangles. Blue triangles indicate the same for the inhibitory input. The peri-stimulus scatter plot (C) demonstrates reliable and precise reproduction of spikes under repeated presentation of such inputs.

The observations on voltage responses of jcBNST neurons under DC current injection might suggest that a history of alcohol dependence has a minor effect on their excitability and firing properties. Indeed, the input resistance, rheobase and the I–N slope parameter were found not statistically different between groups of cells from normal and alcohol dependent rats. Nevertheless, neurons in their natural environment receive synaptic inputs that are stochastic and transient both in timing and amplitude. In such conditions, neuronal excitability, as characterized by evaluating how the firing rate depends on the strength of the excitatory synaptic inputs, might be different from that measured in standard current-step experiments. On the other hand, the neuroadaptive changes induced by alcohol dependence and withdrawal might alter the temporal structure of firing even if the overall excitability of neurons remains unaffected. To address these questions, we performed a comprehensive study of the firing responses of jcBNST neurons subjected to simulated synaptic bombardment. Figure 2 shows the firing response of a type II neuron (Fig. 2A) when receiving synaptic bombardment via dynamic clamp. The neuron fires very reliably and precisely when receiving the synthetic synaptic input in repeated trials as demonstrated by the peri-stimulus scatter plot in Fig. 2C. Spikes in the neuron are triggered by excitatory synaptic current transients while inhibitory inputs modulate their timing reliability and precision.

**Figure 3:**
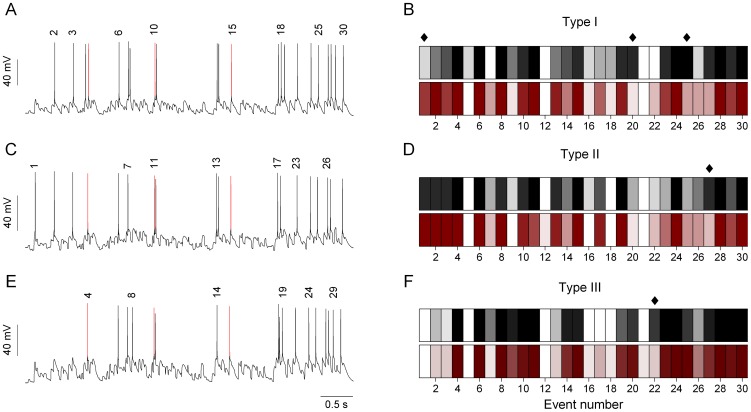
Analysis of firing patterns of jcBNST neurons under dynamic clamp stimulation. The three jcBNST neurons received simulated synaptic bombardment (30 Hz frozen noise template) with the maximal conductances adjusted in a way that each of them emitted approximately 20 spikes per stimulation. Evaluating data from all experiments we found a total of 30 possible locations where spike emission had a non-zero probability. The event numbers are indicated above the spikes in the voltage traces (A, C, E). Events #4, #10 and #15 are indicated by red colored spikes in the 3 cell types. The color-coded histograms on the right (B, D and F) show event occurrence probabilities (reliabilities) for the three cell types using data from both the control (white to black) and the EtOH groups (white to maroon). White bars indicate that no spike was emitted in the corresponding event location. Gradually darker gray (and maroon) colors represent increasing spiking probability in the corresponding events. The 3 cell types can be clearly distinguished upon this analysis, but EtOH treatment has a low impact on the fine structure of responses. Diamond symbols represent over 50% change in spike emission probability (control vs. EtOH comparison). Type I neurons display the highest number (3) of such events.

The cell type-specific expression of voltage-activated ionic conductances result in distinctive features in the voltage responses of jcBNST neurons under DC stimulation. However, as we have shown earlier, these cell type specific intrinsic properties had a relatively weak impact on the firing patterns when the simulated synaptic bombardment was used [39]. The temporal structure of firing patterns appeared qualitatively similar across cell types (Fig. 3A, C, E). Nevertheless, the maximal synaptic conductance that was required to reach a target number of spikes (20) strongly depended on the cell type of the neuron being stimulated. As an example, a type III neuron with relatively low input resistance would need stronger excitatory conductance to emit the target number of spikes than a type II neuron that has higher input resistance and more depolarized resting membrane potential. To quantify the cell type specific differences in the spike responses of jcBNST neurons we constructed histograms that represent firing probabilities in selected event locations along the stimulus sweep (Fig. 3B, D, F). Here, events correspond to single depolarizing current transients (EPSCs) that might be strong enough to bring the neurons membrane potential above firing threshold. When comparing a number of peri-stimulus spike responses from all types of jcBNST neurons, we identified 30 possible event locations where a spike emission was likely at least in a subset of neurons.

While the comparison of the event probability histograms across the 3 types of neurons in normal animals revealed cell type specific differences (although only in a few events), a similar analysis showed more subtle changes when comparing the spike responses of identical types of neurons from control and alcohol dependent animals. A comparison of the pooled firing probabilities showed that the structure of firing was virtually unaffected by alcohol dependence and withdrawal in type II and III neurons (Fig. 3D, F). Type I neurons, on the other hand, displayed a moderate increase of variability in their firing responses during protracted alcohol withdrawal (Fig. 3B). Consequently, alcohol withdrawal in dependent animals has a weak effect on the temporal structure of firing responses under dynamic clamp stimulation. This observation is important, because it indicates that the lack of effect on the firing structure is unlikely due to the choice of the stimulus pattern. We note that during the 5 s of stimulus sweep the instantaneous rate of EPSCs varies in a wide range (cca. 5 to 70 Hz) therefore the jcBNST neurons experience a broadband input. In fact, the repeating 5 s stimulus consists of 150 excitatory and inhibitory current transients each having a different history of preceding input. Assuming that the integrative properties of neurons had changed due to alcohol withdrawal, we would have observed restructuring of the firing patterns, e.g. the arrival of ‘new’ spike events in locations where they were absent in control cells.

### Alcohol withdrawal reduces the dynamic excitability of jcBNST neurons

**Figure 4:**
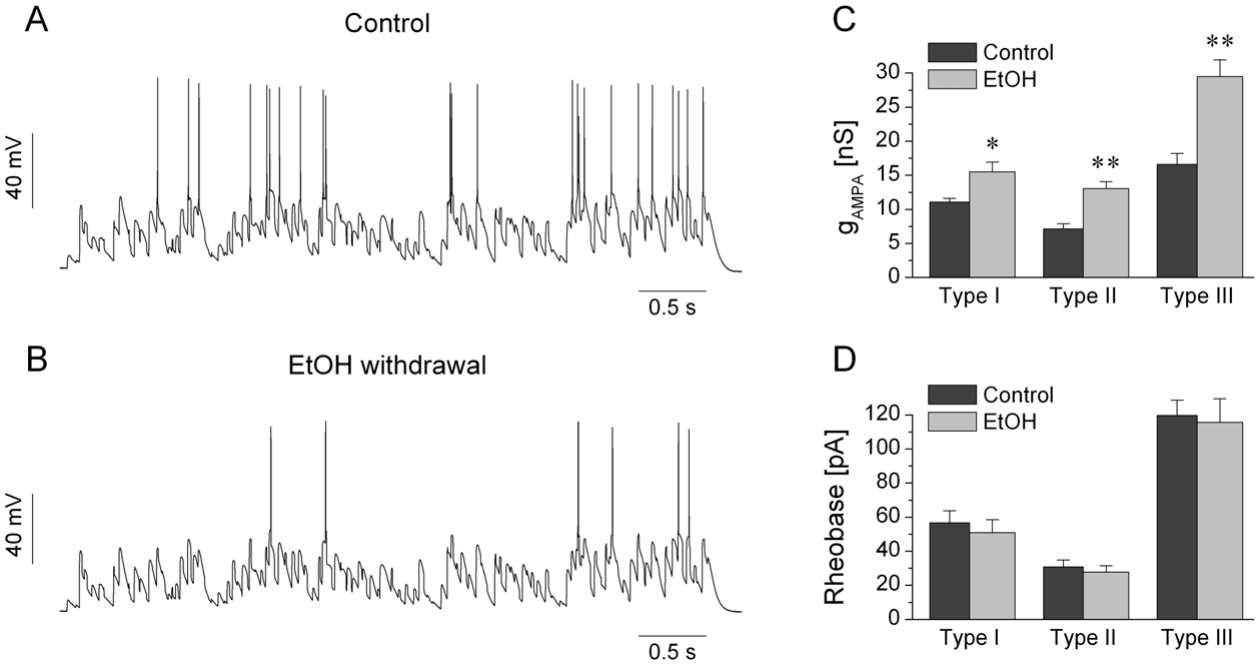
Comparing firing responses of jcBNST neurons under dynamic clamp reveals a strong reduction of excitability of jcBNST neurons from rats under protracted withdrawal from alcohol. Panels A and B demonstrate the firing responses of two type III neurons both receiving identical synaptic stimulation with 30 nS maximal conductance for the AMPA-component. The neuron from the control animal fires intensely (A) while that from the alcohol dependent animal emits far fewer spikes (B). C shows the mean AMPA-conductances that are required to fire a target number (20) of spikes per stimulus sweep. Here, all 3 cell types show a significant increase in the target AMPA-conductance indicating a reduced excitability. Remarkably, the rheobase of firing during DC step stimulation is not significantly altered by alcohol withdrawal (D, see also Table 1.).

As shown above, rheobase of the 3 types of jcBNST neurons (under DC stimulation) differed significantly among cell types suggestive of different degree of intrinsic excitability (Table 1). Among the 3 groups, type III neurons showed the lowest levels of excitability as measured by their rheobase (Table 1). Not unexpectedly, the same neurons required the strongest excitatory synaptic inputs to drive their firing and achieving a selected target number of spikes (20) under one sweep of stimulation in the dynamic clamp setting. Typically, the required maximal conductance (i.e. target conductance) of the excitatory synaptic inputs was more than double for type III than for type II neurons. Hence, the target maximal synaptic conductance can be used as a quantitative measure of excitability of jcBNST neurons under simulated synaptic bombardment. A higher target maximal conductance would indicate lower excitability such as when comparing type III vs. type II neurons. We used this parameter to compare the three cell types as well as the control vs. alcohol dependent groups. Surprisingly, the target maximal conductance of all 3 cell types was markedly increased in the alcohol withdrawal groups relative to the control ones. Fig. 4 demonstrates this effect on a pair of type III neurons and when pooling data from all cells. Here, the voltage traces show the firing responses of a control type III neuron (Fig. 4A) and one from an alcohol dependent animal during protracted withdrawal (Fig. 4B). Here, both neurons received the same noisy conductance waveform and using identical maximal conductances for the AMPA/NMDA/GABA connections (30/30/60 nS). In control the neuron responded with intense firing while the neuron from the dependent animals emitted only 5 spikes per trial. Increasing the maximal conductance of the synaptic inputs by 68% while maintaining the same ratio of AMPA/NMDA/GABA for the latter neuron resulted in firing patterns that were very similar to that of the control neuron, (cumulative data are shown as the bar graphs of Fig. 4C). In fact, all 3 cell types required much stronger maximal conductances in slices from alcohol dependent rats than in control ones. The target maximal AMPA conductances for control type I, II and III cells were 11.1±1.5 nS (n = 11), 7.2±0.7 nS (n = 10) and 16.6±1.6 nS (n = 16), respectively. The same parameters from alcohol dependent slices were 15.5±1.5 nS (n = 13), 13.1±1.0 nS (n = 11) and 29.5±1.8 nS (n = 19), respectively. These observations showed that alcohol withdrawal markedly reduced the dynamic excitability of jcBNST neurons, i.e. their capacity to process incoming synaptic inputs into firing output.

**Figure 5:**
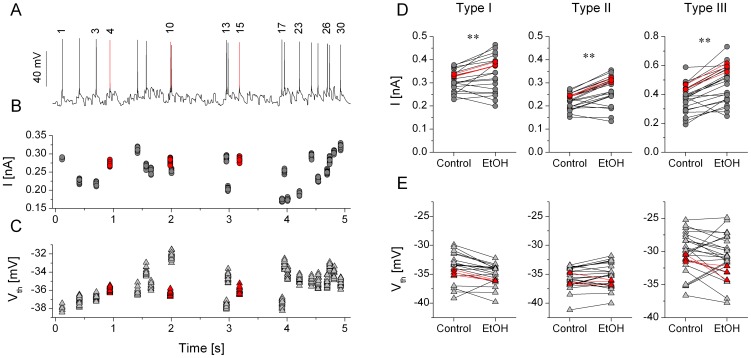
Analysis of spike events reveals a decrease of input resistance near spike threshold of jcBNST neurons from dependent rats. The left panels demonstrate the voltage response of a type II neuron under simulated synaptic bombardment (A) and how the amplitude of the EPSCs (B) and the spike threshold (C) varies along the stimulus. The dynamic clamp stimulus was repeated 20 times. Both the EPSC amplitude and the spike threshold are well reproduced within single spike events, but they vary greatly across events. Paired comparison of mean EPSC amplitudes between control neurons and those from alcohol dependent animals is shown in D. Here, each point represent data from one spike event averaged across cells. All three cell types exhibit a general increase of EPSC amplitude. A similar comparison of spike thresholds reveals no shift (D). Data for event #4, #10 and #15 are indicated with red symbols and the corresponding spikes in A are also colored red.

This observation is interesting because our experiments with constant current stimulation suggested no change in the rheobase or resting input resistance of the neurons due to alcohol withdrawal. However, these 2 parameters characterize the subthreshold and near-threshold behavior of neurons under constant current input and they might be less predictive when applying transient type inputs such as those in our dynamic clamp experiments. In the latter case, every spike is triggered by a brief depolarizing current transient (EPSC) and the spike threshold can also vary in a wider range than in DC step experiments. Hence, we decided to analyze the firing responses on a spike-by-spike basis. Fig. 5 demonstrates how the spike threshold of a type II neuron and the amplitude of the EPSCs vary during repeated presentations of the 5 s of stimulus sweep in our experiments. Repeating the stimulus 20 times we find that both the local EPSC amplitude (Fig. 5B) and the spike threshold (Fig. 5C) are nicely reproduced for the same spike events but vary greatly across events. We note that the conductance waveforms (AMPA-, NMDA- and GABA-type) that are used for the stimulation are identical for each trial, but the current also depends on the postsynaptic membrane potential, therefore it can vary across trials (Fig. 5B). As we showed, the overall pattern of the firing response is remarkably similar between control and alcohol dependent groups of neurons (Fig. 3), hence it is possible to compare the EPSC amplitude and the spike threshold for identical spike locations. Importantly, we found a general shift of EPSC amplitudes for the majority of spike events in all 3 types of neurons when comparing the control and EtOH groups. Fig. 5D shows this comparison for type I, II and III neurons. Here, each point represents the mean of EPSC amplitudes in specific spike locations that are present both in the control and alcohol dependent group of neurons. More than 80% of the EPSC amplitude values are greater in cells of dependent animals than the corresponding values in the controls (all types). The overall shift of EPSC amplitudes is already shown by evaluating the pooled data (see Table 2). Here, the mean EPSC amplitudes for type I, II and III neurons from EtOH animals are consistently higher than those from the controls (p<0.05 in all 3 groups using unpaired t-test). Nevertheless, a paired comparison of EPSC amplitudes for specific spike events is more appropriate for the reasons mentioned above. Here, for all 3 types of neurons we find a uniform increase of EPSC amplitudes at p<0.01 level (Fig. 5D). This analysis clearly shows that jcBNST neurons from animals under protracted alcohol withdrawal require stronger input currents (EPSCs) to reach a target number of spikes than those from control animals. Hence, an increase of the maximal conductance of simulated synaptic connections results in stronger excitatory currents under the dynamic clamp stimulation. One possible reason for this observation might be that the spike threshold of the neurons becomes more depolarized under alcohol withdrawal, therefore stronger excitatory currents are required to drive the firing. Nevertheless, a similar analysis as we did for the EPSC amplitudes reveals no significant increase of spike threshold of jcBNST neurons. A paired comparison of spike threshold levels shows that these are virtually unchanged by alcohol treatment. To summarize these results we find that all three types of jcBNST neurons require stronger synaptic inputs and excitatory postsynaptic currents to reach a target number of spikes, but this effect is not due to a depolarizing shift of their spike threshold (Fig. 5E).

**Table 2:** Comparison of the mean EPSC amplitude and mean spike threshold parameters measured from dynamic clamp experiments.

Cell type	N cells	N events	EPSC amplitude [nA]	Spike threshold [mV]
I _Cont_	8	26	***0.296±0.011*** [0.17 : 0.38]	−33.7±0.5 [−39.2 : −28.4]
I _EtOH_	9	26	***0.352±0.016*** [0.20 : 0.49]	−35.8±0.6 [−42.2 : −31.2]
II _Cont_	5	24	***0.223±0.007*** [0.15 : 0.27]	−35.7±0.4 [−41.2 : −33.4]
II _EtOH_	9	24	***0.256±0.014*** [0.11 : 0.36]	−35.2±0.4 [−40.0 : −31.8]
III _Cont_	9	27	***0.373±0.019*** [0.17 : 0.59]	−30.3±0.5 [−35.2 : −25.2]
III _EtOH_	13	27	***0.503±0.025*** [0.25 : 0.75]	−31.5±0.7 [−41.8 : −24.9]

For each cell type and treatment group the number of cells and number of spike events are shown. In average, each cell produced 20 spikes per stimulus sweep, but the number of possible event locations was higher. Mean EPSC amplitudes are consistently higher for the neurons from the alcohol dependent group (indicated by bold-italic numbers). No significant change is observed in the mean spike threshold. Values are expressed as means ± S.E.M. (unpaired t-test, p<0.05).

## Discussion

In our study we carried out a comprehensive analysis of physiological properties and firing responses of jcBNST neurons under protracted withdrawal from alcohol using two experimental designs. We found that while most physiological parameters of jcBNST neurons were barely affected by alcohol withdrawal in dependent animals, the neurons dynamic excitability as measured from firing responses under the action of barrages of simulated synaptic inputs was markedly reduced.

Synaptic inputs are integrated by and converted into firing of postsynaptic neurons in a way that depends on the operation of multiple voltage-dependent membrane conductances. Clearly, a complex interaction of synaptic inputs and the intrinsic biophysical properties of the postsynaptic neurons results in the firing activity that is the principal function of neurons and neural circuits [40], [41], [42]. Among the several physiological properties of neurons, excitability receives particular attention of investigators, because it indicates how efficiently a specific type of neuron might convert excitatory inputs into the firing output. The excitability depends on several passive and active membrane properties such as the membrane resistance, capacitance, and the amount and kinetics of multiple voltage-gated ionic currents [43], [44]. In our study we characterized the excitability and several other physiological properties of jcBNST neurons using two different experimental protocols, an approach that proved to be valuable for a better understanding of the neuroadaptations induced by a history of alcohol dependence.

Despite the fact that the rheobase measured by DC current injection in our experiments was not affected by a history of alcohol dependence, neuronal excitability as observed from firing responses under the action of barrages of simulated synaptic inputs in the dynamic clamp setting was markedly reduced in all jcBNST neuronal types from dependent rats. This is a novel observation and indicates that adaptive changes in the intrinsic cellular properties of neurons can influence the firing differently under constant current inputs and when the neurons receive more complex conductance inputs that simulate the natural synaptic bombardment. It has been shown that spike threshold strongly depends on the rate of rise of membrane depolarization preceding the spike [43]. Clearly, synaptic inputs *in vivo* result in EPSPs with a wide range of amplitude and slope of membrane depolarization. Thus, spike threshold *in vivo* as well as in dynamic clamp experiments with simulated synaptic bombardment (Fig. 5C) varies in a wider range than in experiments using DC current injection. It is likely that the fluctuating membrane potential that is present under noisy synaptic inputs allows a more complex interplay of the activation and inactivation of multiple voltage-gated membrane conductances thus leading to a more complex regulation of spiking. In such conditions, up- or downregulation of a specific membrane conductance (such as under adaptive changes by alcohol dependence) can result in more significant changes in the neurons firing response than when subjected to a DC stimulus effectively forcing it into a narrower dynamical range.

Our experiments showed that type II neurons that express strong I_h_ in normal animals [37], [38] had weaker voltage sag in neurons from alcohol dependent animals. Downregulation of I_h_ such as we found in type II jcBNST neurons in dependent animals has been also demonstrated in primate inferior olive neurons under protracted withdrawal [11] and in the rat VTA [9]. However, we found marked reductions in excitability not only in type II neurons but also in type III neurons that express no I_h_ and type I neurons that had no significant change in their voltage sag. Additionally, the spike threshold as measured by DC current injection was not significantly affected in type II neurons of alcohol dependent rats (Table I) despite a reduction in the I_h_. We also recognize that due to the activation profile of the I_h_, its influence in regulating (facilitating) the firing is stronger whenever the neurons transiently hyperpolarize to more negative membrane potentials (−70 mV and below). The mean level of membrane potential in our dynamic clamp experiments was in the range of −45 to −40 mV, more depolarized than where the I_h_ supposedly has a significant contribution to the firing. Considering this, even a strong reduction of I_h_ due to neuroadaptation or pharmacological treatment might not alter the firing dynamics of type II neurons at least when receiving AMPA, NMDA and GABA_A_-type synaptic conductances. Indeed, we observed that acute application of the I_h_-blocker ZD7288 under the present simulated synaptic bombardment did not affect the dynamic excitability of type II neurons. Firing responses were compared before and after the application of ZD7288 in dynamic clamp experiments and we found essentially no change in the spike numbers (2.4±3.4% relative change, n = 6). Consequently, the marked reduction of dynamic excitability in alcohol dependent animals which was observed in all 3 cell types suggests that further biophysical mechanisms are involved.

Our event specific analysis of firing responses indicated a general reduction of input resistance of jcBNST neurons near spike threshold when the simulated synaptic bombardment was used. Here, we have to recognize that the input resistance of virtually all jcBNST neurons was a steep function of the injected current, shown by DC step experiments. Indeed, we often observed two-fold or greater increase of input resistance in the −200 to 0 pA range of input current (e.g. Fig. 1J). Similarly, the observed input resistance increased even further when approaching spike threshold (0–50 pA). We note that it becomes increasingly difficult to estimate the input resistance in this range due to the activation of depolarizing membrane conductances. At the same time, a paired comparison of specific spike events in dynamic clamp experiments showed that the spike threshold was not significantly different in jcBNST neurons of control and alcohol-treated animals, although this parameter was varying considerably along the stimulus. This observation suggests that the voltage dependence of the transient Na-current was not altered by protracted alcohol withdrawal in these neurons. However, in neurons from dependent animals stronger excitatory postsynaptic currents were required to reach spike threshold and achieve the target number of spikes. Effectively, this means reduced input resistance of neurons (or increased shunting) near the spike threshold. We can consider alternate mechanisms to explain this finding. For instance, downregulation of a depolarizing current that activates near spike threshold or upregulation of a hyperpolarizing current in the same range would both decrease the net input resistance (i.e. the resistance that reflects a combination of the passive membrane resistance and the contribution of voltage-dependent conductances). The perisomatic Na^+^ persistent current (I_NaP_) is broadly expressed in neurons [45], including in the BNST [38]. Downregulation of this current by protracted alcohol withdrawal might cause adaptations that are consistent with our finding regarding the reduced dynamic excitability of jcBNST neurons. Alternatively, the persistent K-current I_M_ that is active at slightly depolarized membrane potentials has a role in stabilizing the membrane potential in the presence of depolarizing currents [46] hence, upregulation of this current might be consistent with our data.

Another important membrane current that regulates the excitability and spike timing of neurons is the slowly inactivating dendrotoxin-sensitive K-current (I_D_). This current has been shown to delay action potential firing and regulate spike threshold variability in cortical pyramidal neurons [40]. The I_D_ current rapidly activates at near threshold membrane potentials hence it can inhibit the generation of action potentials during fast depolarizing transients like EPSPs (shunting inhibition, [47]). We have previously shown that jcBNST neurons have impaired long-term potentiation of intrinsic excitability (LTP-IE) under protracted withdrawal from alcohol or drugs of abuse [29] and that the loss of LTP-IE of intrinsic excitability was associated with an increased expression of Kv1.2 channels that mediate the I_D_ current [48]. While the baseline excitability of neurons was not significantly altered by alcohol withdrawal in DC step experiments in our previous study [29] and also confirmed by our recent experiments, now we have a clear evidence for reduced dynamic excitability that is revealed by using simulated synaptic bombardment in the dynamic clamp. Thus changes in the I_D_ current may also contribute to the reduced excitability of jcBNST neurons.

In summary, our observations indicate that alcohol withdrawal-related neuroadaptive changes in the intrinsic properties of jcBNST neurons, manifesting as reduced input resistance near spike threshold, effectively decrease their firing responses under excitatory synaptic inputs. This effect on the intrinsic membrane properties might impair synaptic integration in the extended amygdala circuitry potentially reducing the inhibitory regulation of CeA by the BNST. It is also noteworthy that the reduction of neuronal excitability remains largely masked in experiments involving conventional stimulation with DC current injection. Hence, simulation of synaptic conductances with the dynamic clamp technique offers a valuable alternative to study neuronal excitability and functional adaptations.

## Materials and Methods

### Chronic alcohol treatment

All animal protocols were consistent with guidelines issued by the National Institute of Health and approved by our Institutional Animal Care and Use Committee of The Scripps Research Institute (protocol number 07-0068). We used the standard alcohol inhalation method of the Scripps Research Institute Alcohol Research Center to induce ethanol dependence in rats exposed to intermittent alcohol vapors [49]. Wistar rats (Charles River) 2–3 months old at the beginning of the study were made dependent by exposure to air/alcohol vapors in alcohol vapor chambers (La Jolla Alcohol Research, La Jolla, CA) with an intermittent exposure paradigm (14 h on/10 h off) for a total of 4 weeks, which reliably produce alcohol dependence in rats [29], [50]. Intermittent alcohol exposure mimics human patterns of alcohol consumption [49], [51], [52], [53]. Blood alcohol levels were measured with an oxygen-rate alcohol analyzer (Analox Instruments, London, UK) and maintained at (*M* ± SEM) 150±20 mg%. Rats were then used for the study at around 4 weeks withdrawal. Age matched rats non exposed to alcohol vapor but housed in the same caging system and environment and subjected to the same handling were used for controls. Control and dependent animals were kept in identical conditions during the withdrawal period. Previous experiments with control animals has shown that there is no activation of the stress system by housing in the alcohol apparatus measured by plasma ACTH and corticosterone level, indicating the absence of environmental-induced stress in the absence of alcohol [54].

### Brain slices and electrophysiology

Acute brain slices were prepared as previously described [11], [27] with minor modifications. Briefly, coronal rat brain slices (350 µm) were collected from the rostral cerebrum of Wistar rats using a Capden vibrating microtome (Loughborough, England) in oxygenated artificial cerebrospinal fluid (ACSF) consisting of (in mM) 130 NaCl, 3.5 KCl, 24 NaHCO_3_, 1.25 NaH2PO_4_, 2.2 CaCl_2_, 10 d-glucose, and 2 MgSO_4_, pH 7.4. The slices were preincubated in ACSF for 1 hour at 32°C and then maintained at room temperature for at least 30 min before being transferred to a submerged recording chamber. The temperature of the ACSF in the recording chamber was kept 31°C during the recordings and the perfusion was running at 3 ml/min. Slices of brain tissue containing the BNST were placed in a superfusion chamber and visualized with a Leica stereomicroscope under low magnification. Neurons were not visualized during electrode insertion and the experiments (blind recordings). Intracellular current clamp and dynamic clamp experiments were performed in whole-cell configuration using 8–12 MOhm patch pipettes filled with intracellular solution containing (in mM): KMeSO_4_ 120, KCl 10, MgCl_2_ 3, HEPES 10, Phosphocreatine 10, MgATP 2, GTP 0.2; osmolarity set to 280–290 mOsm, pH 7.2. Synaptic isolation of jcBNST neurons was achieved by blocking glutamate and GABA receptors using 10 µM 6-cyano-7-nitroquinoxaline-2,3-dione (CNQX), 50 µM AP-5 and 30 µM bicuculline in the bath. Recordings and intracellular stimulation were made using a Multiclamp 700 amplifier (Axon Instruments) in bridge mode. Stimulus waveforms were generated using the data acquisition software DASYLab 6.0 (Dasytec, Amherst, NH) in a Windows computer equipped with a National Instruments PCI-MIO-16-E4 board. We used standard rectangular current commands as stimuli for the initial physiological characterization of jcBNST neurons. Specifically, we delivered 350 ms pulses of current starting from −200 pA and incremented in 10 pA steps from the resting membrane potential of the cell.

### Dynamic clamp

In addition to the standard current clamp stimulation we elicited firing activity in the jcBNST neurons by stimulating them with a barrage of simulated excitatory and inhibitory synaptic inputs via dynamic clamp [30]. Here, we first generated artificial presynaptic voltage waveforms resembling random firing activity in populations of excitatory and inhibitory neurons [39]. These analog presynaptic waveforms (templates) consisted of 5 ms wide spike-shaped voltage transients that departed from and returned to a rest state of −60 mV (Fig. 2B Exc, Inh). The interspike intervals of both the inhibitory input patterns were Gauss-distributed with a mean of 33 ms and standard deviation of 17 ms. In order to induce postsynaptic currents with variable amplitude we introduced amplitude variation of the spike-shaped voltage transients such that their peak value ranged from −30 to 0 mV in a uniform distribution. In each experiment we used one excitatory and one inhibitory input that were designed using the same statistical parameters but uncorrelated otherwise. The input from the excitatory voltage waveform (Exc) was used to evoke rapid (AMPA-type) and slow (NMDA-type) excitatory conductance transients and postsynaptic potentials (Fig. 2A, B). We used simulated chemical synaptic connections (see [30] for details) to couple the excitatory an inhibitory inputs to the biological neuron. The synaptic time constant (τ_syn_) was 10 and 50 ms for the AMPA- and NMDA-type connections, respectively and the reversal potential (V_rev_) was 0 mV for both. Voltage dependent Mg-block of NMDA receptors was also incorporated in our model in a way that the maximal conductance was a sigmoid function of the postsynaptic membrane potential [55] expressed as
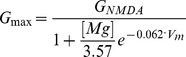
 where [Mg] = 2, expressed in mM and V_m_ was the neurons membrane potential in mV. The second voltage waveform (Inh) served as the GABAergic inhibitory input (V_rev_ = −68 mV, τ_syn_ = 10 ms). The template waveforms were connected to the analog inputs of a Digidata 1200B interface controlled by the dynamic clamp software StdpC v. 1.9. [30] running in a separate computer. We used equal conductances for the two excitatory inputs (AMPA- and NMDA-type) and twice of the excitatory conductance for the GABAergic input (e.g. 5/5/10 nS). Typically, the duration of the random presynaptic waveforms was 5 s (Fig. 2A, C) and they were repeatedly presented every 13 s (frozen noise protocol), hence, the neurons were at rest for 8 s between stimuli. The two synthetic presynaptic voltage waveforms, the injected synaptic current and the voltage output of the biological neuron were acquired simultaneously at 20 kHz sampling rate. To compare spike responses of the 3 jcBNST neuronal types we maintained the spike count constant (mostly 20) among different neurons by adjusting the 3 conductances and maintaining the aforementioned 1/1/2 ratio. Spike emissions in the stimulated neurons were detected on-line (by seeking local maxima of the derivative of membrane potential) and the arrival times were saved into ASCII files. We measured spike arrival times at 50 µs accuracy and in reference to the onset of the stimulus in each trial (sweep). The presynaptic voltage waveforms were generated and the response of the jcBNST neuron was recorded by the DASYLab 6.0 program (Dasytec, Amherst, NH), hence, two separate computers were used for data acquisition and for the dynamic clamp.

### Data analysis

We developed software in CodeGear Delphi 2009 for analyzing voltage responses of the neurons under DC current stimulation. A total of 16 physiological features were obtained from the voltage waveforms including membrane resistance, rheobase, time constants of the voltage sag among others (see Results). We obtained two input resistance curves measured either from points at the local maxima of the voltage deflection (R_max_) or 10 ms before the end of the hyperpolarizing current steps (R_end_) (Fig. 1B, F, J). The slope of the resistance curves as obtained by linear fitting of the points between −0.1 and 0 nA was used as a quantitative measure of inward rectification (for type III neurons where the I_KIR_ conductance is abundant). The slope of the voltage sag curve under hyperpolarizing current steps was used as also used as a distinctive measure of cell types I and II. The 16 physiological parameters were compared across three cell types and two treatment groups of animals (i.e. controls vs. EtOH withdrawal) using one-way ANOVA and two-sample t-tests, respectively. Firing patterns obtained in the dynamic clamp experiments were initially analyzed using peri-stimulus scatter plots. Reliable spike events in such plots manifested as vertically aligned tick marks, i.e. when a spike was emitted repeatedly in the same location of the stimulus. Reliability for each spike event was calculated by counting the trials with successful spike emission and dividing this count by the total number of trials. Reliability values for single spike events were averaged across cells of the same type to construct event probability histograms.
